# Low blue carbon storage in eelgrass (*Zostera marina*) meadows on the Pacific Coast of Canada

**DOI:** 10.1371/journal.pone.0198348

**Published:** 2018-06-13

**Authors:** Victoria R. Postlethwaite, Aimee E. McGowan, Karen E. Kohfeld, Cliff L. K. Robinson, Marlow G. Pellatt

**Affiliations:** 1 Resource and Environmental Management, Simon Fraser University, Burnaby, British Columbia, Canada; 2 Marine Protected Areas Research Group, University of Victoria, Victoria, British Columbia, Canada; 3 Protected Areas Establishment and Conservation Directorate, Parks Canada, Vancouver, British Columbia, Canada; University of Waikato, NEW ZEALAND

## Abstract

Seagrass habitats provide important ecosystem services, including their ability to take up and store substantial amounts of organic carbon, known as ‘blue carbon.’ However, the paucity of geospatial and carbon storage information along the Pacific Coast of Canada hinders the inclusion of blue carbon storage data in conservation planning and policy development in coastal habitats. We assessed the carbon storage and accumulation rates in three eelgrass (*Zostera marina*) meadows in southern Clayoquot Sound on the Pacific Coast of British Columbia. The intertidal and subtidal portions of each meadow were mapped and sampled to estimate eelgrass density, biomass, and carbon, and sediment cores were analyzed to estimate sediment carbon storage and accumulation rates. Aboveground biomass measurements were consistent with estimates for *Z*. *marina* in other regions, with average aboveground carbon biomass estimates of 16.78 g C m^-2^ and 16.25 g C m^-2^ in the intertidal and subtidal areas, respectively. However, the estimated aboveground to belowground biomass ratio was an order of magnitude higher than for seagrass species in temperate/tropical areas, largely because belowground biomass was up to 10 times lower than for other *Z*. *marina* meadows, averaging 6.17 g C m^-2^ and 5.03 g C m^-2^ in the intertidal and subtidal zones, respectively. Sediment carbon concentrations did not exceed 1.30%C_org_, and carbon accumulation rates ranged from 2.90–39.61 g C_org_ m^-2^ yr^-1^, decreasing with depth and averaging 10.8 ± 5.2 g C_org_ m^-2^ yr^-1^. While sediment carbon stocks were generally higher in the eelgrass meadows relative to non-vegetated reference sites, carbons stocks averaged 1343 ± 482 g C_org_ m^-2^, substantially less than global averages. These carbon results confirm that eelgrass does contribute to carbon storage in Clayoquot Sound but at lower rates than identified for more tropical seagrasses. By improving the quantification of site-specific carbon dynamics, eelgrass’ role in climate change mitigation and conservation planning can be assessed.

## Introduction

Coastal marine habitats are recognized as highly productive ecosystems worldwide. However, their potential to accumulate and store organic carbon (C_org_), known as ‘blue carbon’, has largely been overlooked on the Pacific Coast of North America (in the Northeast Pacific seagrass bioregion) [1, 2]. Vegetated coastal ecosystems, such as mangroves, salt marshes, and seagrass meadows, have been estimated to bury carbon at a higher rate per unit area than terrestrial forests, and store a disproportionate amount of carbon for their relatively small area [2]. Recent assessments suggest 177,000–600,000 km^2^ of Earth’s total area is covered in seagrass habitats, potentially storing 48–112 Tg C per year [2]. This carbon storage amount is approximately 10% of the yearly total organic carbon burial in the ocean, even though seagrass meadows occupy less than 0.2% of the total ocean area [1].

Along the Pacific Coast of Canada and the United States, the eelgrass *Zostera marina* is a commonly distributed seagrass located in shallow areas of temperate estuaries [3]. In addition to eelgrass’ role in ecosystem regulation, sediment deposition, and substrate stabilization, *Z*. *marina* and other eelgrass species are thought to act as an important carbon sink [1, 3]. This carbon is stored in the sediment, accumulating mainly from *in situ* production and sedimentation processes [4]. Additionally, seagrass meadows, due to water inundation, are predominantly anaerobic, slowing microbial carbon oxidation and release [5]. The sediment can also accrete vertically for a longer period of time than terrestrial forests, increasing carbon accumulation [6].

Agriculture, forestry, and commercial developments have destroyed substantial amounts of seagrass habitat along the British Columbia (BC) coastline [7], which has likely affected blue carbon sequestration in BC. Previous studies have shown that conservation of marine vegetated ecosystems results in greater carbon storage capacity than restoration of degraded habitats [8]. However, given the paucity of geospatial information on blue carbon environments, the rate of seagrass habitat loss in coastal BC is uncertain [9]. This lack of data hinders the incorporation of carbon management into coastal conservation planning and policy development on the Pacific Coast of Canada [10, 11]. Furthermore, uncertainties in the carbon storage capacities of seagrass meadows in different environments have led to major generalizations regarding global blue carbon stocks [12]. Most seagrass data are derived from tropical-subtropical regions and used to extrapolate worldwide blue carbon estimates [13]. Providing adequate data on regionally specific seagrass meadows could rectify this oversimplification in global blue carbon calculations. Improved quantification of regional blue carbon dynamics could help determine eelgrass’ role in mitigating climate change [6, 13], as well as its potential for use in carbon markets (e.g. [14]).

Efforts to assess seagrass distribution, extent, density, and blue carbon storage potential have been initiated in North America (NA) to develop more effective strategies for seagrass habitat protection and management [3, 15]. To date, 47,775 km^2^ of seagrass meadows are thought to exist across NA, yet only 24,190 km^2^ have been mapped [16]. Given the lack of geospatial information on the extent, area, and carbon dynamics of seagrass meadows, the Pacific Coast of NA has recently been identified as a high priority location for seagrass mapping and carbon sequestration research [17].

This study quantitatively assessed above- and belowground biomass, carbon storage, and carbon accumulation rates within the intertidal and subtidal zones of three eelgrass meadows on the Pacific Coast of Canada. This research fills the data gap in the Northeast Pacific seagrass bioregion on the extent, density, and biomass of *Z*. *marina* and provides regional carbon stock estimates for biomass and sediment in eelgrass along coastal BC. We compare these carbon storage and accumulation rates with global estimates that are based largely on measurements from tropical regions, as well as with other *Z*. *marina* meadows.

## Site location

**Fig 1:**
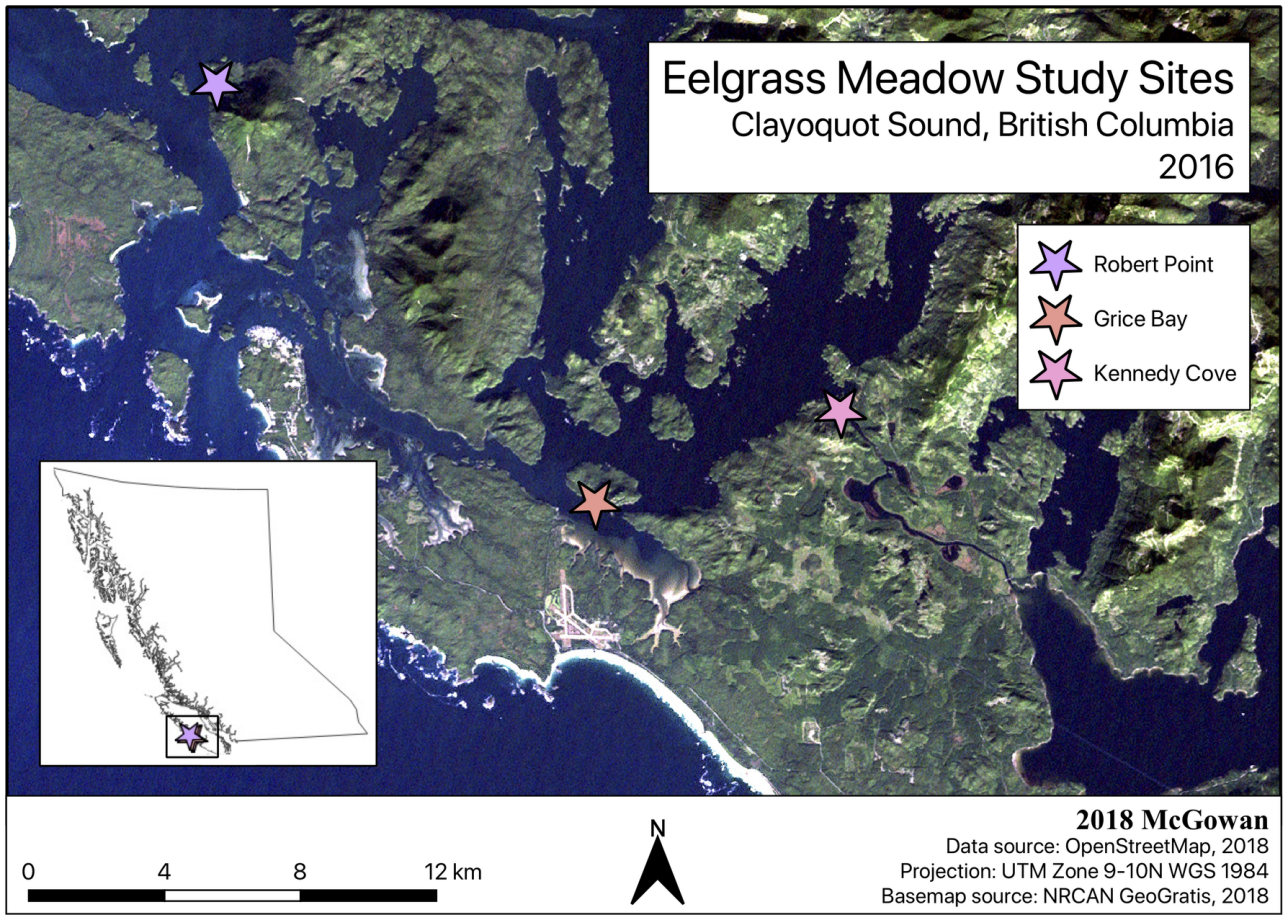
Location of eelgrass meadows sampled in Clayoquot Sound, British Columbia.

The three study sites, Robert Point, Grice Bay, and Kennedy Cove, are located within Tofino Inlet in the southern region of Clayoquot Sound (CS), British Columbia (Fig 1). Grice Bay is also situated within the Long Beach Unit of Pacific Rim National Park Reserve of Canada. The eelgrass meadows were selected for two reasons: first, the meadows are in regions with low human population and thus experience fewer anthropogenic effects. Second, the meadows are distributed along a gradient in water temperature and surface salinity, as a result of freshwater discharge from the lower Kennedy River. Many variables have been shown to influence carbon sequestration, including meadow nutrient regime, sediment trapping, sediment export, and water column irradiance; the selected study meadows represent a wide range of these variables [18–20]. Robert Point, our most marine site and furthest from the lower Kennedy River (~22km), had a salinity of 29.3 ± 0.5, and Grice Bay (~8.5 km from the river) had a slightly lower salinity at 24.1 ± 0.1. Kennedy Cove, adjacent to the lower Kennedy River, has much more freshwater influence, with a salinity of only 6.3 ± 0.4. Surface water temperatures were lowest at Robert Point (12.9 ± 0.1 °C), closely followed by Grice Bay (13.9 ± 0.1 °C), and warmed to 18.1 ± 0.1 °C at Kennedy Cove. Secchi disc measurements indicated that water column irradiance was lowest at Robert Point (4.8 ± 0.1 m), followed by Grice Bay (6.0 ± 0.5 m) and Kennedy Cove (7.7 ± 0.1 m).

## Materials and methods

### Eelgrass meadow mapping

The areal extent of the intertidal and subtidal regions of each meadow was estimated in ArcGIS v 10.3 [21] using: 1) GPS coordinates to demarcate the intertidal and subtidal zones; 2) underwater videos to delineate boundaries of the subtidal zone, and 3) quadcopter aerial images to generate large-scale, regional maps of each meadow. Data were collected at the Robert Point and Grice Bay meadows during low tide periods (0.5m) on 25–26 May 2016, and at the Kennedy Cove meadow during the lowest low tide (0m) on 6 June 2016.

Intertidal boundaries were defined by walking along the landward edge to the seaward edge of each meadow during low tide; GPS waypoints were collected every 3–7 metres. The upper-intertidal edge was defined as the point 0.5 m past the last shoot within the meadow; this method recognizes that roots can extend a fair distance from an individual shoot [22]. The intertidal-subtidal division was also walked during low tide, and GPS coordinates were collected to delineate the low intertidal from shallow subtidal. Subtidal surveying was completed by towing an underwater high definition video camera along the subtidal edge of each meadow [7, 22]. GPS coordinates were marked every 3–7 seconds and labeled with a descriptor (e.g., outer subtidal edge, patchy area, bare spot). The subtidal edge was identified using methods of [7] and [23], i.e., when density decreased below 1 shoot m^-2^ and shoots were more than 1 m^2^ away from one another.

A DJI Phantom 3 professional quadcopter, Map Pilot App, and Maps Made Easy software were used to create high resolution GeoTIFFs of the Robert Point and Kennedy Cove meadows. Grice Bay was not flown over because it was located adjacent to the Tofino airport. The quadcopter was flown above the meadows during low tide, capturing images which were georeferenced using the Phantom’s FC300X built-in camera. Maps Made Easy software geo-stitched the aerial images to produce a high resolution GeoTIFF. The aerial images and geospatial data were uploaded into ArcGIS v 10.3 [21] to determine the distribution, extent, and area of the intertidal and subtidal regions of each meadow. The data were then exported into Q GIS 3 [24] to design the maps of each meadow.

### Eelgrass biomass collection and living carbon analyses

At each meadow during low tide, three 50 m transects were placed parallel to the beach.

Transect A was representative of the upper-intertidal; Transect B was representative of the low-intertidal; and Transect C represented the shallow subtidal. Due to the nature of the Kennedy Cove meadow (i.e. fringe, narrow bed), only two transects were set: an intertidal (Transect A) and subtidal (Transect C).

Twelve 0.25 m^2^ quadrats were placed along each transect, and the distance between them was set using a random number generator. A total of 36 samples were collected from each meadow: 24 from the intertidal and 12 from the subtidal. At Kennedy Cove, 18 quadrats were laid along each of the two transects. A GPS waypoint and photograph were collected at each quadrat to facilitate mapping each biomass sample site. The number of eelgrass shoots within each quadrat were counted, and the length of 5 blades from 5 different shoots were measured to the nearest mm [7, 25].

A 5 cm diameter and 10 cm deep core (0.002 m^2^) was used to collect eelgrass biomass samples from each 0.25 m^2^ quadrat [23, 25]. Cores were removed using a simple push coring method. The shoots in each core sample were counted, and the length and width of the longest blade from at least 4 shoots within each sample were recorded to the nearest mm; width measurements were recorded at 1 cm above the sheath [23]. Algal epiphytes were scraped off the blades, and the relative abundance (%) of each type of epiphyte was estimated [26, 27]. Leaf blades and sheaths were separated from roots and rhizomes to estimate the aboveground biomass (AGB) and belowground biomass (BGB) for each core [25]. Epiphytes, aboveground biomass, and belowground plant material were placed into a drying oven at 60°C for 24 hours [26, 27], and the dry material was weighed.

The AGB and BGB measurements from each 0.002 m^2^ core were upscaled to represent 1 m^2^ to compare to other studies. To estimate AGB, dry weight biomass per shoot was determined for each core by dividing the aboveground dry weight of shoots and leaves (DW) by the total number of shoots in that core (S_C_) [22, 28]. AGB for each 0.25 m^2^ quadrat was then determined by multiplying the shoot dry weight by the number of shoots in its respective quadrat (SQ) [29], and then scaled up to represent biomass per square meter by dividing the weight of each 0.25 m^2^ quadrat by its area (A_Q_) [25]. The same calculation was applied to the BGB (roots and rhizomes of each core):

$$B=(\frac{DW}{S_{c}}.SQ)÷A_{Q}$$ (1)

where B = aboveground or belowground biomass.

A standard conversion factor for *Z*. *marina*, 0.36, was used to calculate the amount of carbon (g C m^-2^) in AGB and BGB [30]. While the value of 0.34 is sometimes used for this conversion [6], we chose to use a value of 0.36 because it more specifically relates to habitats dominated by *Z*. *marina*. Average intertidal and subtidal AGB and BGB (g DW m^-2^) estimates were derived for each transect in the study meadows; Transects A and B were then pooled to provide biomass and living organic carbon estimates for the entire intertidal portion of each meadow.

### Sediment core collection and carbon analyses

Three sediment cores were collected along each of Transects A and C in the upper-intertidal and shallow subtidal zones (S1 Table). One “reference” core was also collected ~250 m from each meadow in three non-vegetated areas. Reference sites were selected to have similar substrate and exposure but with no eelgrass present. The large area of the Grice Bay eelgrass meadow made it difficult to find a suitable reference site, and thus the reference core was collected near the meadow (~50 m). Cores were taken using a simple push method, where three-inch polycarbonate tubes were bevelled on one end to help cut through eelgrass shoots and rhizomes in the top layer of sediment, and then pushed into the sediment until depth of refusal. Although recent standard practice for carbon analysis in seagrass recommends sediment cores of 1 m length [6], the sediment at these study sites did not allow for extraction of 1 m cores, and therefore ‘depth of refusal’ was used. This method resulted in minimal (<2 cm) compaction. Cores were extracted in the field at 1 cm intervals into sterile sample bags and were handled carefully so as not to disturb soil compaction, allowing for accurate dry bulk density (DBD) measurements. The bags were kept in coolers until they were brought back to the laboratory and refrigerated at 4°C.

In the laboratory, DBD measurements were taken at each 1 cm interval for each core by sampling a known volume of sediment, drying the sediment at 60°C for no less than 96 hours, and weighing the sediment to obtain g cm^-3^ values [6]. Loss-on-ignition (LOI) was performed on every 1 cm subsample by removing roots or rhizomes, drying a small amount of sediment (<5g), weighing the dry sample, and then combusting it for 4 hours at 550°C, followed by re-weighing, and obtaining the weight difference from combustion.

In a small subset of samples, organic carbon content was also determined by measuring the total carbon (%TC) and inorganic carbon (%IC) contents in the same samples, using CHN Elemental and coulometric analysis, respectively. Measurements of %IC were subtracted from the %TC measurements to estimate %C_org_ [15]. The values for %IC ranged from 0.0019 to 0.0544% and did not substantially contribute to the sediment carbon content. Gravel particles (>2mm) were not able to be ground, and therefore were removed prior to carbon analyses. The percent of gravel, found through grain size analysis and averaged for every 5 cm, was subtracted from uncorrected %C_org_ values to obtain true %C_org_ values. The % gravel content was only substantial in the Kennedy Cove cores, where % gravel content ranged from 2 to 43%, and therefore significantly reduced initial %C_org_ results at this site.

Measurements of %C_org_ from elemental analysis (EA) were related to measurements of weight %LOI made on the same subset of samples using a simple linear regression (S1 Fig). The resulting regression equation was then applied to each weight % LOI value to estimate %C_org(LOI)_ in all samples where %C_org_ had not been estimated using elemental analysis. Carbon density (g C_org_ cm^-3^) was estimated for each 1-cm sample interval by multiplying the DBD (g cm^-3^) and %C_org_ fraction (%C_org_/100); carbon stocks (g C_org_ cm^-2^) were then totalled over the length of each core, which ranged from 24 cm (Kennedy Cove intertidal) to a maximum length of 51 cm (Robert Point intertidal). Carbon stocks were scaled up to represent a square metre to allow comparison with other studies.

Subsamples from nine cores, including the three reference cores, were sent for ^210^Pb analysis, to obtain radiometric dates for determining mass accretion and carbon accumulation rates. Between 5 and 18 ^210^Pb measurements were made on each core by Core Scientific International, Winnipeg, Canada, who used a ^210^Pb constant rate of supply model [31] to construct age-depth relationships and determine sediment mass accretion rates (cm yr^-1^). From mass accretion rates, sediment accumulation rates (SAR) were calculated using dry bulk density, and then carbon accumulation rates (CAR) were determined by multiplying the C_org_ fraction (%C_org_/100) by SAR.

### Statistical analyses

One-way ANOVAs were used to test the differences in aboveground and belowground biomass, biomass carbon, and carbon stocks and accumulation rates within sites (intertidal v. subtidal) and between the three sites and reference sites. The significance level of these tests was set at α = 0.05, and the data were log-transformed to meet the parametric assumptions of an ANOVA when necessary. Assumptions of normality were tested by examining residuals of the data. Statistical analyses were performed in R [32].

## Results

### Eelgrass meadow surface area

**Fig 2:**
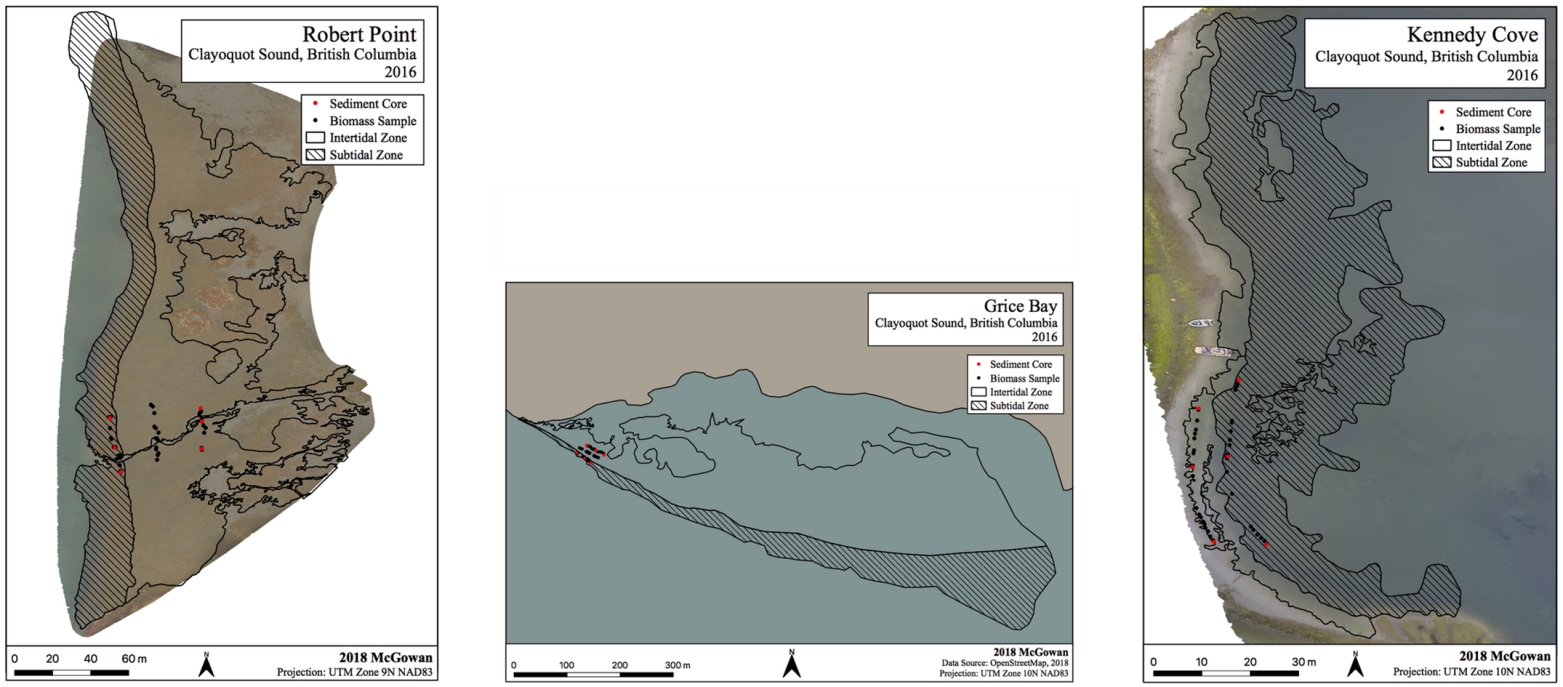
Extent of intertidal and subtidal zones in eelgrass meadows at Robert Point (left), Grice Bay (middle) and Kennedy Cove (right). Intertidal and subtidal meadows are differentiated by hashed and blank patterns, respectively. Black dots: quadrats for biomass collection, red dots: sediment core locations.

The eelgrass meadow furthest from the lower Kennedy River, Robert Point, had a total surface area of 31,900 m^2^ (3.19 ha), of which the intertidal component was estimated at 22,400 m^2^ (2.24 ha; 70%). The Grice Bay meadow, located within the Pacific Rim National Park Reserve and mid-way from the lower Kennedy River, had a total surface area of 262,000 m^2^ (26.20 ha), 71% (18.60 ha) of which was intertidal. The smallest eelgrass meadow, Kennedy Cove, closest to the lower Kennedy River and had a total surface area of 5,340 m^2^ (0.53 ha), with a relatively small intertidal component (0.10 ha; 19%) (Fig 2).

### Eelgrass density, biomass and living organic carbon estimates

Average shoot densities ranged from 45 ± 16 shoots m^-2^ in the subtidal zone of Kennedy Cove to highest values of 272 ± 67 shoots m^-2^ in the lower-intertidal zone of Grice Bay. At all three meadows, no significant differences were found in shoot densities between the intertidal and subtidal zones. In all three meadows, mean blade lengths were highest in the subtidal zones, and ranged from 496 ± 240 mm in the upper-intertidal zone at Grice Bay to maximum estimates of 898 ± 257 mm in the subtidal zone at Robert Point (S2 Table).

**Fig 3:**
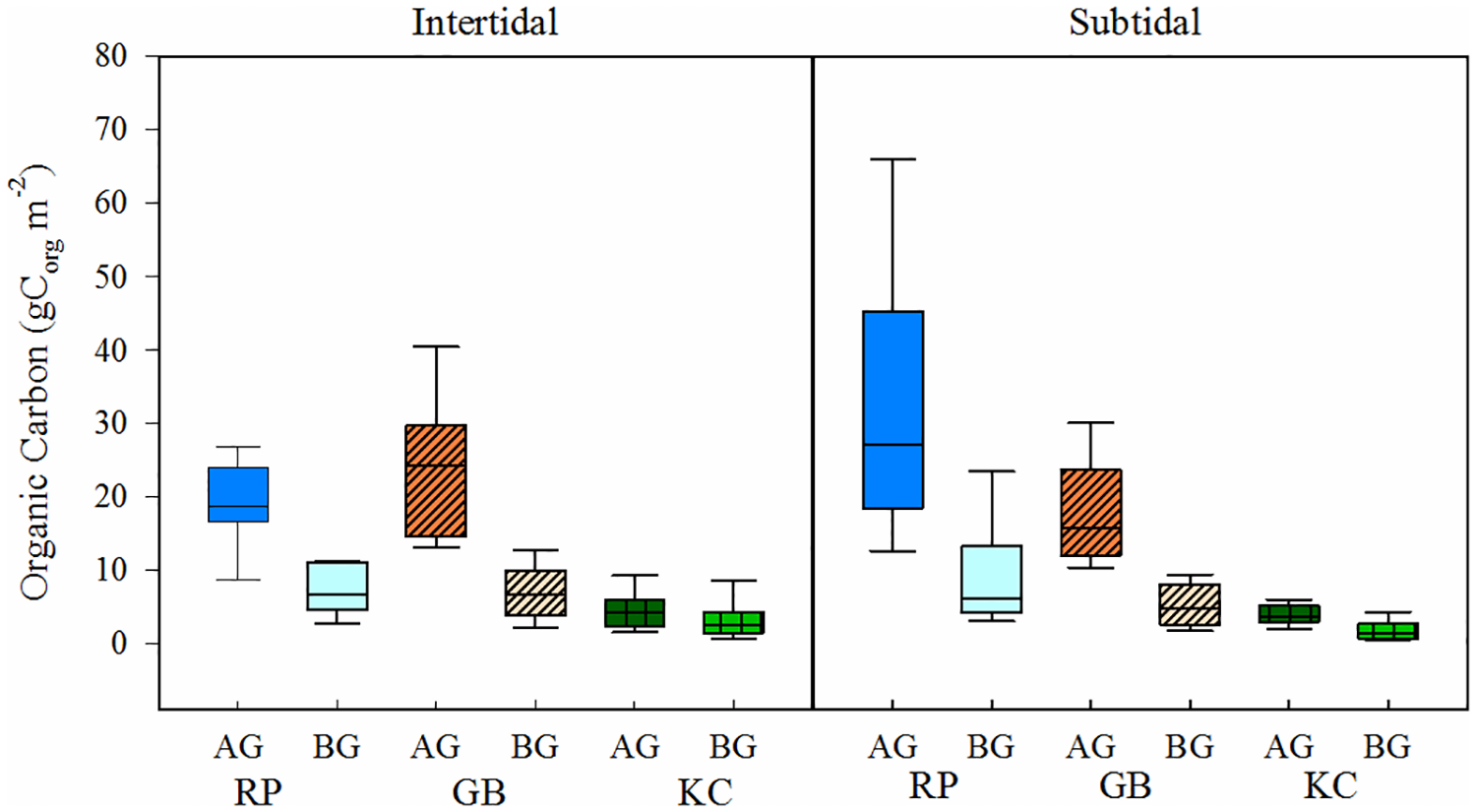
Aboveground and belowground biomass carbon in Robert Point, Grice Bay, and Kennedy Cove. AG: aboveground, BG: belowground, RP: Robert Point meadow, GB: Grice Bay Meadow, KC: Kennedy Cove meadow.

In the intertidal meadows, average AGB ranged from 13 ± 8 g DW m^-2^ at Kennedy Cove to 65 ± 47 g DW m^-2^ at Grice Bay, which translated into carbon stocks of 5 ± 3 to 23 ± 17 g C m^-2^ (Fig 3; S3 Table). In the subtidal meadows, average AGB ranged from 11 ± 4 g DW m^-2^ at Kennedy Cove to 92 ± 51 g DW m^-2^ at Robert Point, which translated into carbon stocks from 4 ± 1 to 33 ± 18 g C m^-2^. There were no significant differences in AGB or aboveground carbon between the intertidal and subtidal portions of the meadows; however, Robert Point and Grice Bay had significantly higher AGB and aboveground carbon than the Kennedy Cove meadow (p<0.001), and Robert Point had slightly higher AGB and aboveground carbon in the subtidal meadow than Grice Bay (p = 0.039).

Estimates of BGB and belowground carbon storage were substantially lower than aboveground (Fig 3). Average BGB in the intertidal zone ranged from 10 ± 8 g DW m^-2^ at Kennedy Cove to 20 ± 17 g DW m^-2^ (essentially the same at Robert Point and Grice Bay), which translated into carbon stocks of 4 ± 3 to 7 ± 6 g C m^-2^. In the subtidal meadows, BGB ranged from 5 ± 4 g DW m^-2^ (or 2 ± 1 g C m^-2^) at Kennedy Cove to 26 ± 20 g DW m^-2^ (or 9 ± 7 g C m^-2^) at Robert Point. Similar to aboveground, there were no significant differences in the BGB or belowground carbon between the intertidal and subtidal portions of each meadow; however, Robert Point and Grice Bay had significantly higher BGB and belowground carbon than the Kennedy Cove meadow (p<0.001). Robert Point and Grice Bay were not statistically different.

### Sediment properties, carbon stocks, and accumulation rates

The sediment at Grice Bay and Robert Point was predominantly sand with a small proportion of mud. A thick shell layer was present at the bottom of each core in these two meadows, as is common in most seagrass sediment [17]. Kennedy Cove’s sediment was predominantly mud and gravel, and no shell fragments were present.

DBD values were not significantly different at Robert Point and Grice Bay, ranging from 1.05 to 1.25 g cm^-3^ (n = 213) for Robert Point and from 0.94 to 1.30 g cm-3 (n = 239) at Grice Bay. DBD values for Kennedy Cove were significantly higher (p<0.001) than Robert Point and Grice Bay (0.84–1.68 g cm^-3^, n = 173), due to heavy pieces of gravel (S4 Table).

Percent C_org_ was low (<1.30%) and declined with depth at all three sites. Percent C_org_ was lowest (<1%) in Robert Point (n = 213) and Grice Bay (n = 239), ranging from 0.02–0.82%C_org_, and highest at Kennedy Cove where values ranged from 0.15–1.29%C_org_ (n = 173). All sites were statistically different from each other (p<0.001), with Robert Point having the lowest values. The only significant differences between subtidal and intertidal carbon concentrations were found at Grice Bay where percent C_org_ was significantly higher in the subtidal meadow (p = 0.005). The Robert Point and Kennedy Cove meadows had significantly higher carbon stocks than their associated reference sites, whereas the Grice Bay meadow did not.

**Fig 4:**
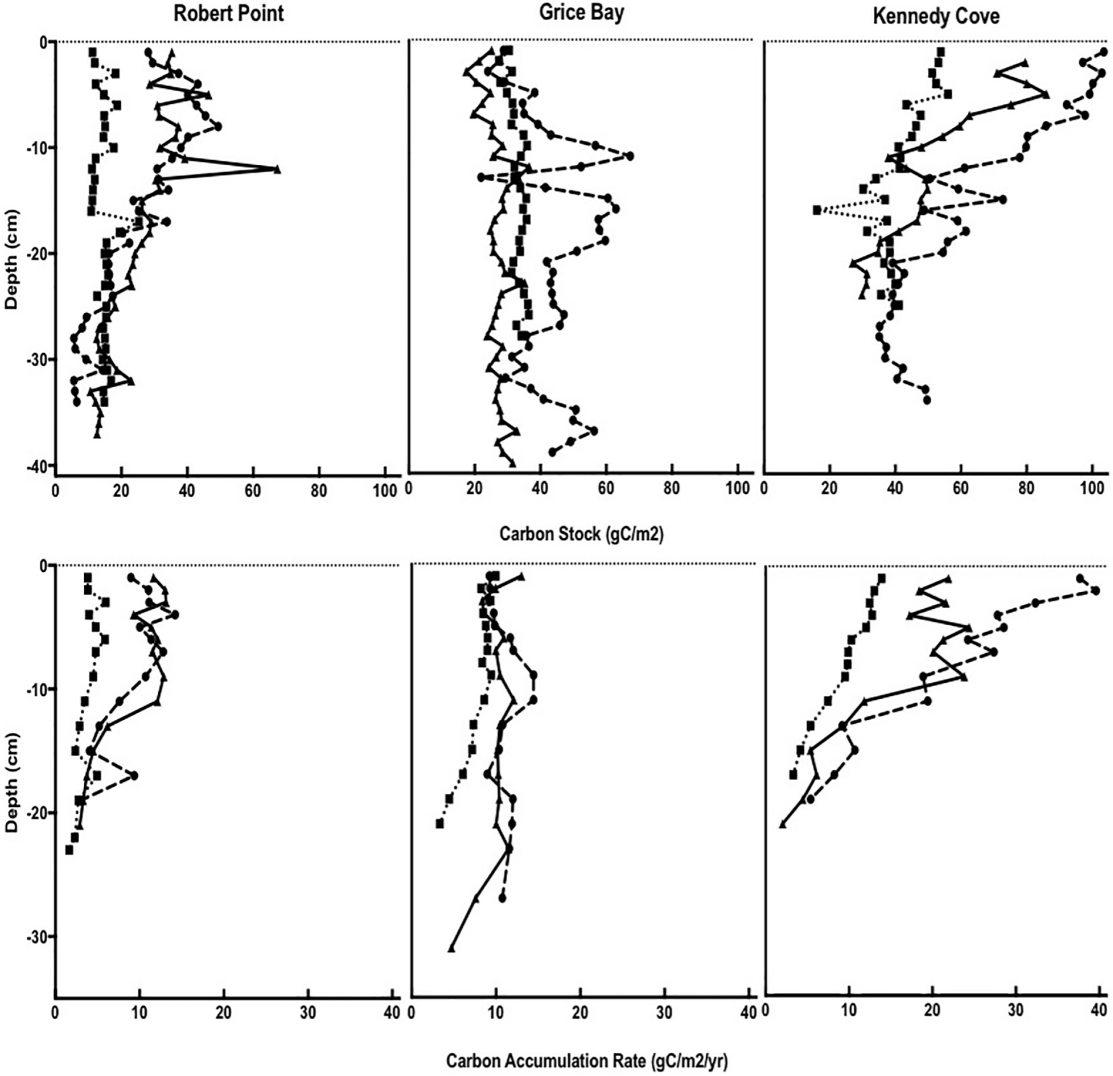
Carbon stock and accumulation rates in Robert Point, Grice Bay, and Kennedy Cove. Solid lines: intertidal, dotted lines: subtidal, dashed lines: reference sites.

Overall, sediment carbon stocks (incorporating depth) averaged 1343 ± 482 g C_org_ m^-2^ for the region, ranging from lowest average values of 820 ± 26 g C_org_ m^-2^ at Robert Point’s subtidal meadow (34 cm average depth) to highest average values of 2099 ± 365 g C_org_ m^-2^ at Kennedy Cove’s subtidal meadow (34 cm average depth) (Fig 4). Carbon stocks were lowest in the more marine-based Robert Point meadow (802–1166 g C_org_ m^-2^, 30-51cm depth), slightly higher at Grice Bay (947–1924 g C_org_ m^-2^, 34-47cm depth), and highest near the mouth of the Kennedy River at Kennedy Cove (979–2519 g C_org_ m^-2^, 21–39 cm depth). Like the percent C_org_, all sites were significantly different from each other, and the Robert Point and Kennedy Cove meadows also had significantly higher carbon stocks than their associated reference sites, whereas the Grice Bay meadow did not. However, the reference site at Grice Bay was difficult to find due to the vast seagrass meadow and therefore located close to the meadow itself. Thus, underground carbon transport may be responsible for the carbon stock of the Grice Bay reference site. Grice Bay had significantly higher carbon stocks in the subtidal meadow compared to the intertidal (p<0.001).

SAR per cm depth averaged 3021 ± 549 g m^-2^ yr^-1^ for all three meadows. SARs ranged from 1036–3674 g m^-2^ yr^-1^ at Robert Point, 2553–4908 g m^-2^ yr^-1^ at Grice Bay, and 841–3140 g m^-2^ yr^-1^ at Kennedy Cove. CAR averaged 10.8 ± 5.2 g C_org_ m^-2^ yr^-1^ for all meadows, with higher rates near the surface and declining with depth (Fig 4). CARs ranged from 2.9–14.2 g C_org_ m^-2^ yr^-1^ at Robert Point, from 4.7–13.0 g C_org_ m^-2^ yr^-1^ at Grice Bay, and were highest at Kennedy Cove where they ranged from 2.1–39.6 g C_org_ m^-2^ yr^-1^. Additionally, the sediments were relatively young. The ^210^Pb was undetectable past 31cm, and the ages for these depths did not exceed 125 years (S5 Table).

**Fig 5:**
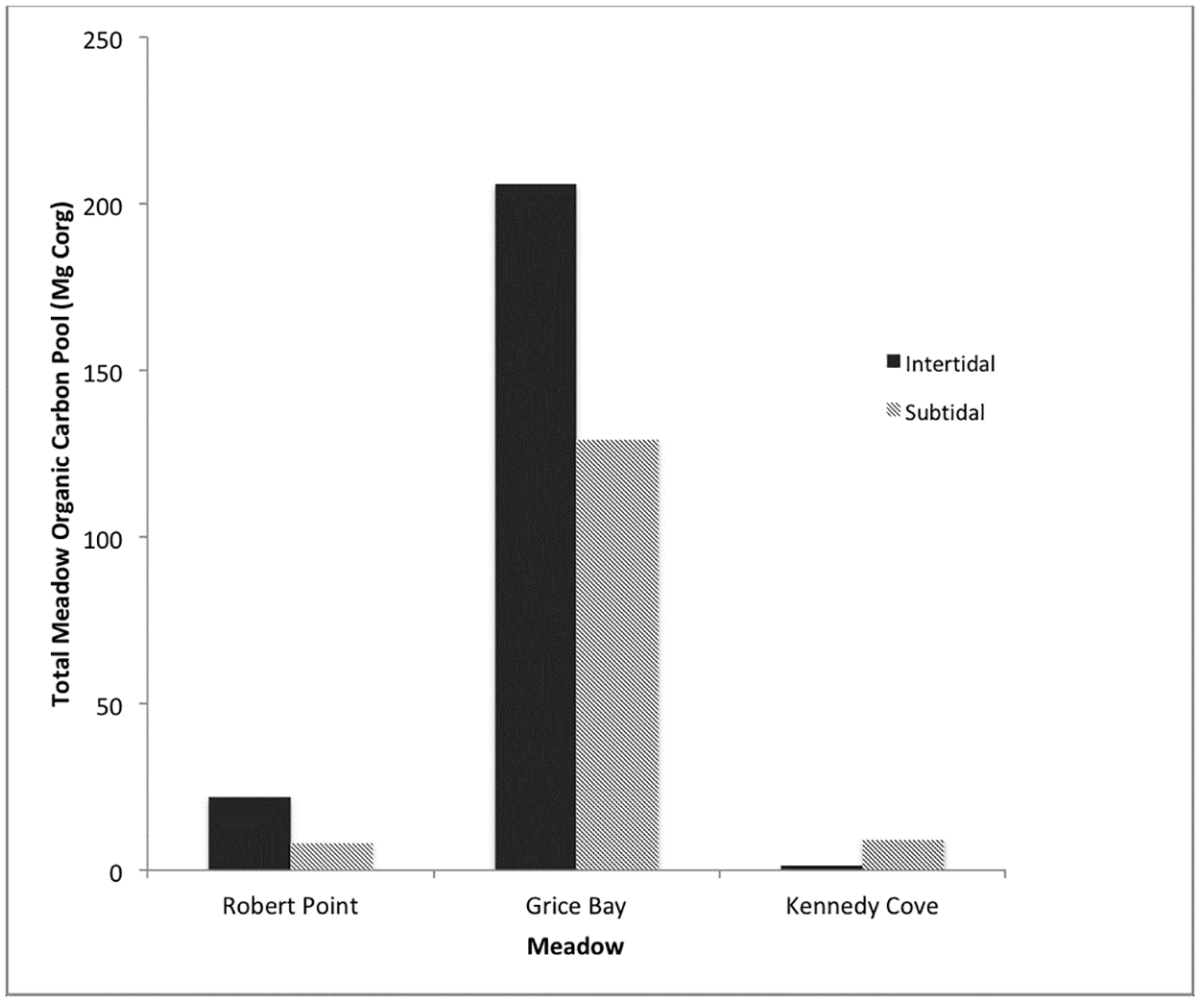
Carbon pool in the total intertidal and subtidal meadows of Robert Point, Grice Bay and Kennedy Cove, from aboveground biomass, belowground biomass, and sediment.

While the sediment carbon content per unit area was highest at Kennedy Cove, Grice Bay sediments contained the most carbon after accounting for the size of the meadow (262,000 m^2^, as compared to 31,900 m^2^ at Robert Point and 5340 m^2^ at Kennedy Cove). Total meadow sediment carbon content was calculated by determining the average depth of refusal for each meadow and zone, and calculating carbon content to that depth. When sediment, aboveground, and belowground carbon contents were combined, Grice Bay had the highest total carbon of 335.9 ± 38.7 Mg C_org_, due to its vast size. Robert Point had the second largest total carbon stock at 30.2 ± 3.1 Mg C_org_ and the smallest, most freshwater, and warmest site, Kennedy Cove, had the smallest total carbon pool at 10.4 ± 1.5 Mg C_org_ (Fig 5).

## Discussion

This study provides the first estimates of biomass, carbon storage, and carbon accumulation for the intertidal and subtidal components of eelgrass meadows in Clayoquot Sound, BC. In terms of biomass, our AGB estimates of 11 ± 4 to 92 ± 51 g DW m^-2^ are in keeping with other *Z*. *marina* meadows, which range from 5 to 145 g DW m^-2^ [33–35]. In contrast, the intertidal and subtidal BGB estimates for the Clayoquot Sound meadows were ~10 times lower than values for *Z*. *marina* found in other regions, where estimates range from 49–244 g DW m^-2^ [34, 36, 37]. The ratio of AGB:BGB was substantially higher in the Pacific Coast meadows when compared with AGB:BGB estimates for *Z*. *marina* and other species of seagrass. While our estimates of AGB:BGB ranged from 1.7–4.3, BGB was found to be 2–3 times *greater* than AGB in other regions [35, 38].

The low values of BGB in Clayoquot Sound are likely a response to (1) sub-optimal light conditions, and (2) possible nitrogen limitation in the growing season. In southern California, previous work [35] has found that greater hours of light saturated photosynthesis (8h vs 4h) promoted the generation of larger roots/rhizomes to store photosynthates, as BGB accounts for most of the soluble carbohydrates in *Z*. *marina*. Sunlight hours (where sunlight reaches the ground) in southern California averages ~9h per day [39], whereas in Tofino Inlet in Clayoquot Sound, BC, sunlight hours are only 5.37h on average [40], showing that light availability is reduced in this region compared to other populations of *Z*. *marina*. Previous research [35] also found that lower light levels were related to a greater shoot length, likely due to eelgrass elongating their leaves towards the water’s surface to reach light, again highlighting the reason for the large difference between AGB and BGB. We saw a similar pattern between our sites, with Robert Point having the lowest secchi disc depth at 4.8 ± 0.1m but the highest blade lengths, averaging at 702 mm. Kennedy Cove experienced the deepest light levels (secchi disc depth of 7.7 ± 0.1 m) but shortest blades (average 660 mm), as well as significantly lower AGB and BGB than Robert Point and Grice Bay, demonstrating that light availability is a driver of variability even within one region.

A second contributor to low BGB may be nitrogen limitation. Seagrasses require a high volume of inorganic nutrients due to their high rates of primary productivity, meaning that they can often be nutrient limited [41]. Seagrass tissue nitrogen content is highly variable, and tends to be highest in winter and lowest in summer [25, 42]. A median N content of 1.8% (of dry weight) has been used to discriminate between nutrient limited and nutrient sufficient ecosystems [25]. The median nitrogen concentration of the sediment in our eelgrass meadows was only 0.041% (S6 Table), indicating nitrogen limitation in the summer months. Biomass responses to nitrogen enhancement or limitation are species specific [43]; *Z*. *marina* shows minor productivity increases in the roots and rhizomes [44]. This indicates that low BGB may be impacted by nitrogen limitation but is likely not the only factor. This same slight increase in productivity was not noticed in the leaf tissue [44], which explains why the AGB biomass in our study did not appear to be affected by nutrient limitation.

Carbon concentrations, stocks, and accumulation rates in Clayoquot Sound were all substantially lower than estimates from other regions. While carbon stocks ranged from 802–2519 g C_org_ m^-2^ in Clayoquot Sound eelgrass meadows, recent global estimates average 19,420 ± 2020 g C_org_ m^-2^ [1]. For *Z*. *marina*, estimates from the Baltic Sea and Northern Europe range from 500–4324 for the top 25 cm [34, 45], placing our findings within the range of carbon stocks for *Z*. *marina* but much lower than global averages. Similarly, the sediment carbon contents (% C_org_) estimated in this study are much lower than those found in the literature. Values from Australia, the Mediterranean, and Florida range from 0 to 48% C_org_, with an average value of 2.5 ± 0.1% C_org_ [1, 10]. Carbon values in this study did not exceed 1.30% C_org_. Finally, carbon accumulation (sequestration) rates in this study were very low as compared to previously published estimates. While CAR ranged from 2.05–39.61 g C_org_ m^-2^ yr^-1^ and averaged 10.8 g C_org_ m^-2^ yr^-1^ in Clayoquot Sound, global estimates range from 45–190 g C_org_ m^-2^ yr^-1^ and average 138 g C_org_ m^-2^ yr^-1^ [2]. *Z*. *marina* estimates of CAR range from 0.84–36.68 [4, 13, 46], indicating our values are in line with other estimates for *Z*. *marina* but lower than global averages. Recent studies from British Columbia have also found similarly low values, indicating that low carbon stocks are likely a regional feature [15, 47].

Four main factors may contribute to the low carbon stocks in this study, including the (1) shallow root system of *Z*. *marina*, (2) sediment type, (3) depth over which sedimentation and carbon accumulation occurred, and (4) low sediment discharge to the meadows from terrestrial sources. First, as discussed above, the species *Z*. *marina* tends to have much shallower roots than *P*. *oceanica* and other Mediterranean species [5], which produce thick root mats that promote sediment carbon storage [48]. The shallow root system of *Z*. *marina* are likely not sufficient to trap carbon through sedimentation processes, resulting in low carbon storage [49]. Additionally, the highly patchy nature of eelgrass meadows in Clayoquot Sound is a likely contributor to lower carbon stock estimates [50].

A second contributor to the low carbon stocks may be sediment type. Fine grained particles, such as mud, tend to promote more carbon adsorption [51]. The Robert Point and Grice Bay meadows, in particular, contained a large proportion of sand. Previous studies found the lowest carbon stocks for *Z*. *marina* at sandy, exposed sites in the Baltic Sea [34]. The large range of carbon stock estimates found in *Z*. *marina* studies [13, 34] suggests a large dynamic range in carbon stocks found in *Z*. *marina* meadows. Our results support recent conclusions that current seagrass carbon stocks might be overestimated due to the lack of data on specific meadow characteristics and fluxes [49, 50].

The third factor contributing to low carbon stocks is the shallow depth of sedimentation in the Clayoquot Sound meadows. Sediment core depths ranged between 21 and 51cm, below which a thick gravel or shell hash layer was found. Additionally, dates from ^210^Pb analysis show that sediments have not been accumulating for very long (~100 years at ~20 cm depth). This shallow sedimentation depth and short accumulation time may be explained by tectonic uplift of the Pacific Northwest [52]. The Clayoquot Sound region is experiencing uplift at a rate of 2.6 mm per year, comparable to current global rates of sea level rise [52]. Regional uplift is likely not allowing thick beds to form, reducing long-term carbon storage. Additionally, the Tofino Inlet has an average annual precipitation of 3305 mm [40], which is significantly more than other, more well-documented blue carbon eelgrass studies like Florida (~1500 mm) [53], the Mediterranean Sea (variable but averages do not exceed 1400 mm) [54], and Australia (<550 mm) [55]. Thus, regional storm activity in Clayoquot Sound may prohibit long-term sediment accumulation.

Finally, low sediment discharge may be contributing to the low sediment accumulation and allocthonous contribution to the carbon stock. This low sediment discharge can be seen through relatively high secchi disc measurements (4.8–8.8 m) and C:N ratios indicative of predominantly marine influence [56]. Previous research [56] estimates that C:N ratios of <12 indicate marine sources, and we found a mean C:N ratio of 9.54 ± 2.19. Limited terrestrial sources of sediment may indicate low sediment discharge into the meadows, possibly resulting in low sediment accumulation and, when combined with low autochthonous carbon input, low carbon accumulation. The sites, like the majority of eelgrass meadows, including those with *Z*. *marina* [57], also exist in relatively sheltered areas, which may influence the low carbon accumulation rates. Further isotopic data are needed to confirm marine and terrestrial inputs into the meadows.

We note that we did not follow previous recommendations to extrapolate cores down to 1 m when estimating carbon stocks and accumulation rates [1, 17]. The thick shell hash layer at depth of refusal in our sediment cores coupled with declining CARs indicated that In our sediment cores, CARs decrease to less than 0.15 g C m^-2^ yr^-1^ after 25 cm depth, so assuming carbon accumulation to 1 m would have grossly over-estimated carbon stocks for our sites. Using a 1 m standard has received some criticism in the literature recently [49] because of the likelihood of overestimating carbon stocks in some systems. However, other studies have noted that extrapolating the carbon stocks in short cores to 1 m actually underestimated the total carbon stocks found in associated, deeper cores [1]. Overall, this suggests depth of carbon accumulation has high geographic variability.

Our results suggest very few significant differences in biomass and carbon between the intertidal and subtidal meadows, where the only difference was that the Grice Bay subtidal meadow had significantly higher carbon concentrations and stocks than those found in the intertidal meadow. This may be due to erosion by wave action disrupting carbon storage within the intertidal meadow, whereas in the fully inundated areas of the meadow (ie. subtidal), decay rates are likely slower, increasing the carbon storage [2]. However, this trend was not seen in the other meadows. While other studies have found a negative relationship between carbon storage and increasing water depth [13], our results show few if any differences in carbon storage between the subtidal and intertidal zones. However, we only sampled the shallow subtidal zone, and more substantial differences in C_org_ storage may be seen if deep subtidal meadows had been sampled. Significant differences in carbon storage were also absent between intertidal and subtidal meadows for Australian meadows of *Posidonia sinuosa* and *P*. *australis* [5].

While eelgrass in the Clayoquot Sound appears to have low C_org_ values, carbon stocks were significantly higher in the eelgrass meadows compared to the non-vegetated reference sites, which has been seen in previous studies [50]. This confirms that eelgrass does contribute to carbon storage in CS but not at rates identified for more tropical seagrasses.

While differences between the intertidal and subtidal meadows were limited, our results suggest strong spatial patterns. Overall, the highest total combined (sediment and living biomass) carbon contents were found in the two meadows with high salinity and cooler water (Grice Bay, closely followed by Robert Point), with the least amount of carbon in the warmer, less saline Kennedy Cove meadow. These differences in carbon stock are likely related both to meadow size and shoot density, as well as the meadow’s environmental characteristics. First, on an aerial basis, Grice Bay’s large intertidal meadow had 10–20 times the carbon storage capacity compared to Kennedy Cove or Robert Point because of the size of the meadow. The Kennedy Cove meadow also had substantially lower aboveground biomass and carbon contents when compared with the other two meadows, likely related to lower shoot densities in the intertidal zone. Eelgrass is known to increase sedimentation [4], and thus high carbon storage in large flats (e.g., Grice Bay) may be due to their increased capacity for sedimentation of fine mud particles. Fringe meadows, such as Kennedy Cove, are narrow and are less able to capture sediment.

Secondly, the differences in environmental characteristics, such as salinity and temperature, play an important role. *Z*. *marina* occurs throughout the northern hemisphere in a wide range of salinities between 3 and 30 [58]. Physiological stress symptoms, including reduced photosynthetic capacity, have been seen at salinities <9 [59], which may be a reason for decreased eelgrass health at Kennedy Cove (6.3). Different eelgrass populations, however, show marked adaptability to salinity levels, and so there are likely additional factors affecting the Clayoquot Sound eelgrass, such as temperature. *Z*. *marina* in the Pacific Northwest are healthiest at 5–8°C, and exhibit physiological stress at temperatures above 15°C [60]. Therefore, Kennedy Cove, with a water temperature of 18.1°C (and presumably warmer later in the summer), is likely too warm in the growing season for the eelgrass to thrive, resulting in low shoot densities compared to the other, cooler meadows. Spatial variability clearly plays an important role in eelgrass biomass and carbon storage, and temporal factors are likely another driver in these ecosystems. Further investigation into the factors influencing variability in eelgrass meadows is needed.

This study emphasizes the need for regionally specific data to ground-truth global estimates. Coastal ecosystems, including eelgrass meadows, are highly variable and their structure is dependent on a variety of environmental factors, meaning that extrapolation from global averages has led to overestimation of blue carbon in some regions. The improved quantification of site-specific carbon dynamics, like those of this study, helps determine eelgrass’ role in climate change mitigation and potentially their use in carbon markets across the world.

## Conclusion

This study quantified above and belowground biomass and carbon storage and accumulation rates within the intertidal and subtidal footprints of three seagrass meadows on the Pacific Coast of Canada.

Aboveground biomass was within the range of global estimates, ranging from 11 ± 4 g DW m^-2^ (4 ± 1 g C m^-2^) at Kennedy Cove to 92 ± 51 g DW m^-2^ (33 ± 18 g C_org_ m^-2^) at Robert Point. Belowground biomass, however, was significantly lower than expected, ~10 times lower than values for *Z*. *marina* in other regions, ranging from 5 ± 4 g DW m^-2^ (2 ± 1 g C m^-2^) at Kennedy Cove to 26 ± 20 g DW m^-2^ (or 9 ± 7 g C m^-2^) at Robert Point. AGB:BGB (shoot:root) was also significantly higher than global estimates. This disparity between above and below ground biomass is likely a response to sub-optimal light conditions and nitrogen limitation.

The three sites had significantly lower carbon storage than previous estimates from global studies, ranging from 10 ± 1.5 Mg C_org_ at Kennedy Cove to 335.9 ± 38.7 Mg C_org_ at Grice Bay. Sediment was the main contributor to carbon storage, with an average of 122.7 Mg C_org_ per meadow stored in the sediment, compared to 2.1 and 06 Mg C_org_ for AGB and BGB, respectively. Carbon accumulation rates were also significantly lower than global estimates but in line with preliminary regional estimates and other estimates for *Z*. *marina*. Low carbon storage is likely a result of 1) *Z*. *marina*’s shallow root system, 2) the highly patchy nature of the eelgrass meadows, 3) sandy sediment at the sites, and 4) the shallow depth of sedimentation.

Eelgrass meadows in Clayoquot Sound, and potentially in the Pacific Northwest, may not store as much carbon as tropical meadows, but generally had a larger carbon stock than the reference cores, meaning that the eelgrass meadows still contribute to carbon storage and could be used in climate change mitigation strategies. It is crucial to protect these ecosystems to protect their carbon sequestration capacity, as well as preserve the ancillary ecosystem services eelgrass meadows provide.

## Supporting information

S1 Fig EA %C_org_ vs. weight % LOI regression analysis, with an R^2^ value of 0.74. EA: elemental analysis, C_org_ = organic carbon, LOI: loss-on-ignition.
(TIFF)

S1 Table Location of cores collected in the intertidal and subtidal of each eelgrass meadow sampled. IT: intertidal, ST: subtidal, RP: Robert Point, GB: Grice Bay, KC: Kennedy Cove.
(DOCX)

S2 Table Average number of shoots per 1-m^2^ and blade length at Robert Point, Grice Bay and Kennedy Cove. SD: standard deviation.
(DOCX)

S3 Table Aboveground and belowground biomass in the intertidal and subtidal meadows at Robert Point, Grice Bay, and Kennedy Cove. IT: intertidal, ST: subtidal, AGB, aboveground biomass, BGB: belowground biomass, DW: dry weight, Avg: average, SD: standard deviation.
(DOCX)

S4 Table Sediment carbon and accumulation results for the intertidal and subtidal meadows at Robert Point, Grice Bay, and Kennedy Cove. IT: intertidal, ST: subtidal, DBD: dry bulk density, C_org_: organic carbon, Avg: average. SAR: sediment accretion rate, CAR: carbon accumulation rate.
(DOCX)

S5 Table Ages of sediment in Robert Point, Grice Bay, and Kennedy Cove, in the intertidal, subtidal, and reference sites, estimated using the ^210^Pb constant rate of supply (CRS) model. RP: Robert Point, GB: Grice Bay, KC: Kennedy Cove, IT: intertidal, ST: subtidal, NS: not sampled.
(DOCX)

S6 Table Average nitrogen content (%N) and carbon:nitrogen ratio (C:N) of sediment in the intertidal and subtidal zones of Robert Point, Grice Bay and Kennedy Cove. SD: standard deviation, DW: dry weight.
(DOCX)
