# Supplementary figures and images for: Low blue carbon storage in eelgrass (Zostera marina) meadows on the Pacific Coast of Canada
Source: PLoS One. 2018 Jun 13;13(6):e0198348. doi: 10.1371/journal.pone.0198348 (PMC5999096; doi:10.1371/journal.pone.0198348)

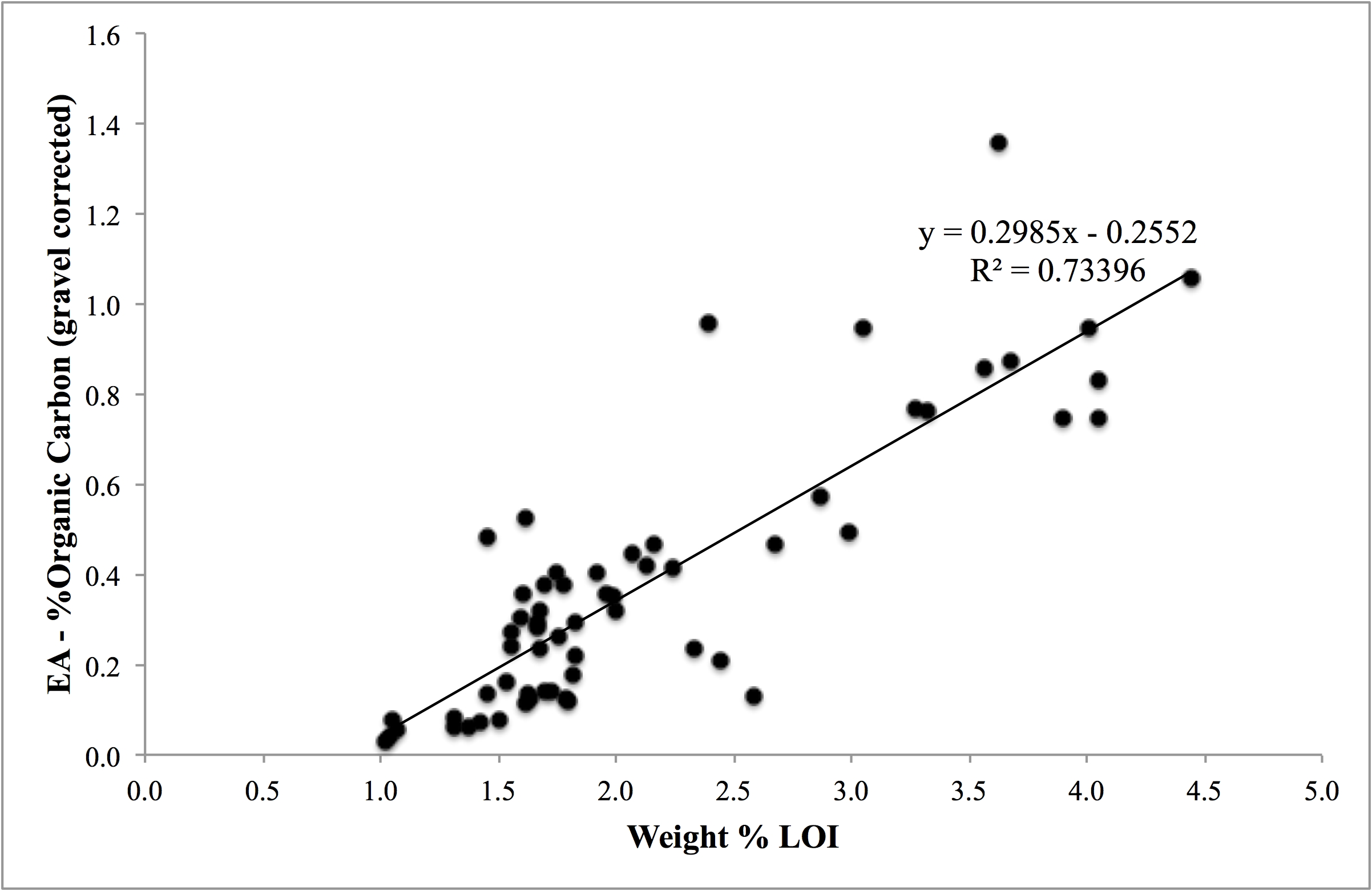

Supplement: S1 Fig — EA: elemental analysis, Corg = organic carbon, LOI: loss-on-ignition. (TIFF) [file pone.0198348.s001.tiff]
