# Supplementary material for: Low blue carbon storage in eelgrass (Zostera marina) meadows on the Pacific Coast of Canada
Source: PLoS One. 2018 Jun 13;13(6):e0198348. doi: 10.1371/journal.pone.0198348 (PMC5999096; doi:10.1371/journal.pone.0198348)
Supplement: S1 Table — IT: intertidal, ST: subtidal, RP: Robert Point, GB: Grice Bay, KC: Kennedy Cove. (DOCX) [file pone.0198348.s002.docx]

**S1 Table. Location of cores collected in the intertidal and subtidal of each eelgrass meadow sampled.** IT: intertidal, ST: subtidal, RP: Robert Point, GB: Grice Bay, KC: Kennedy Cove

| **Core Number** | **Latitude** | **Longitude** | **Date Collected** | **Core Length (cm)** |
| --- | --- | --- | --- | --- |
| **Robert Point** | | | | |
| RP 1 IT | 49.13064°N | 125.55835°W | 24-05-2016 | 51 |
| RP 2 IT | 49.13071°N | 125.55829°W | 24-05-2016 | 31 |
| RP 3 ST | 49.13070°N | 125.55836°W | 24-05-2016 | 40 |
| RP 4 ST | 49.13063°N | 125.55815°W | 24-05-2016 | 35 |
| RP 5 ST | 49.21782°N | 125.93055°W | 26-05-2016 | 31 |
| RP 6 IT | 49.21781°N | 125.92999°W | 26-05-2016 | 30 |
| RP Reference | 49.11057°N | 125.56418°W | 27-05-2016 | 33 |
| **Grice Bay** | | | | |
| GB 1 IT | 49.06747°N | 125.46528°W | 25-05-2016 | 40 |
| GB 2 ST | 49.06743°N | 125.46535°W | 25-05-2016 | 34 |
| GB 3 ST | 49.08368°N | 125.43713°W | 25-05-2016 | 43 |
| GB 4 IT | 49.06731°N | 125.46509°W | 25-05-2016 | 47 |
| GB 5 IT | 49.06743°N | 125.46516°W | 25-05-2016 | 35 |
| GB 6 ST | 49.06731°N | 125.46499°W | 26-05-2016 | 40 |
| GB Reference | 49.07299°N | 125.48171°W | 08-06-2016 | 28 |
| **Kennedy Cove** | | | | |
| KC 1 ST | 49.08340°N | 125.49481°W | 07-06-2016 | 30 |
| KC 2 ST | 49.08422°N | 125.49481°W | 07-06-2016 | 32 |
| KC 3 ST | 49.08337°N | 125.49487°W | 07-06-2016 | 39 |
| KC 4 IT | 49.08297°N | 125.40472°W | 06-06-2016 | 27 |
| KC 5 IT | 49.08299°N | 125.40475°W | 06-06-2016 | 24 |
| KC 6 IT | 49.08309°N | 125.40475°W | 06-06-2016 | 20 |
| KC Reference | 49.08768°N | 125.40156°W | 07-06-2016 | 24 |
