# Supplementary material for: Low blue carbon storage in eelgrass (Zostera marina) meadows on the Pacific Coast of Canada
Source: PLoS One. 2018 Jun 13;13(6):e0198348. doi: 10.1371/journal.pone.0198348 (PMC5999096; doi:10.1371/journal.pone.0198348)
Supplement: S2 Table — SD: standard deviation. (DOCX) [file pone.0198348.s003.docx]

**S2 Table. Average number of shoots per 1-m^2^ and blade length at Robert Point, Grice Bay and Kennedy Cove.** SD: standard deviation

|  | Shoot Count | | Blade Length (mm) | |
| --- | --- | --- | --- | --- |
| Zone | Average | SD | Average | SD |
| **Robert Point** | | | | |
| Transect A | 155 | 95 | 538 | 132 |
| Transect B | 256 | 80 | 671 | 165 |
| Transect C | 254 | 96 | 898 | 257 |
| Grice Bay | | | | |
| Transect A | 99 | 77 | 496 | 240 |
| Transect B | 272 | 67 | 745 | 203 |
| Transect C | 131 | 44 | 724 | 156 |
| Kennedy Cove | | | | |
| Transect A | 54 | 20 | 578 | 165 |
| Transect C | 45 | 16 | 743 | 176 |
