# Supplementary material for: Low blue carbon storage in eelgrass (Zostera marina) meadows on the Pacific Coast of Canada
Source: PLoS One. 2018 Jun 13;13(6):e0198348. doi: 10.1371/journal.pone.0198348 (PMC5999096; doi:10.1371/journal.pone.0198348)
Supplement: S3 Table — IT: intertidal, ST: subtidal, AGB, aboveground biomass, BGB: belowground biomass, DW: dry weight, Avg: average, SD: standard deviation. (DOCX) [file pone.0198348.s004.docx]

**S3 Table. Aboveground and belowground biomass in the intertidal and subtidal meadows at Robert Point, Grice Bay, and Kennedy Cove.** IT: intertidal, ST: subtidal, AGB, aboveground biomass, BGB: belowground biomass, DW: dry weight, Avg: average, SD: standard deviation

| **Zone** | **Average (g DW m^-2^)** | **SD (g DW m^-2^)** | **Range (g DW m^-2^)** |
| --- | --- | --- | --- |
| **Robert Point** | | | |
| IT AGB | 53 | 25 | 9-106 |
| IT BGB | 20 | 14 | 0.05-56 |
| ST AGB | 92 | 51 | 31-195 |
| ST BGB | 26 | 20 | 8-70 |
| **Grice Bay** | | | |
| IT AGB | 65 | 47 | 9-157 |
| IT BGB | 20 | 17 | 2-68 |
| ST AGB | 49 | 20 | 27-84 |
| ST BGB | 15 | 8 | 4-26 |
| **Kennedy Cove** | | | |
| IT AGB | 13 | 8 | 4-31 |
| IT BGB | 10 | 8 | 1-33 |
| ST AGB | 11 | 4 | 5-17 |
| ST BGB | 5 | 4 | 1-5 |
