# Supplementary material for: Low blue carbon storage in eelgrass (Zostera marina) meadows on the Pacific Coast of Canada
Source: PLoS One. 2018 Jun 13;13(6):e0198348. doi: 10.1371/journal.pone.0198348 (PMC5999096; doi:10.1371/journal.pone.0198348)
Supplement: S4 Table — IT: intertidal, ST: subtidal, DBD: dry bulk density, Corg: organic carbon, Avg: average. SAR: sediment accretion rate, CAR: carbon accumulation rate. (DOCX) [file pone.0198348.s005.docx]

**S4 Table. Sediment carbon and accumulation results for the intertidal and subtidal meadows at Robert Point, Grice Bay, and Kennedy Cove.** IT: intertidal, ST: subtidal, DBD: dry bulk density, C_org_: organic carbon, Avg: average. SAR: sediment accumulation rate, CAR: carbon accumulation rate

| **Core ID** | Avg DBD  (g cm^-3^) | Avg  **%C_org_** | Carbon Stock   (g C_org_ m^-2^) | Age at Max  ^210^Pb Depth  (years before June 2016) | Avg SAR  (g m^-2^ yr^-1^) | Avg CAR  **(g C_org_ m^-2^ yr^-1^)** |
| --- | --- | --- | --- | --- | --- | --- |
| **Robert Point** | | | | | | |
| Intertidal | 1.15 ± 0.04 | 0.24 ± 0.13 | 955 ± 138 | 114.5 | 2633 ± 888 | 9.12 ± 4.04 |
| Subtidal | 1.16 ± 0.02 | 0.21 ± 0.12 | 820 ± 26 | 90.8 | 2808 ± 711 | 9.20 ± 3.01 |
| Reference | 1.14 ± 0.02 | 0.13 ± 0.03 | 503 | 104.1 | 3272 ± 646 | 3.87 ± 1.31 |
| **Grice Bay** | | | | | | |
| Intertidal | 1.14 ± 0.06 | 0.24 ± 0.04 | 1074 ± 186 | 94.3 | 4291 ± 644 | 9.92 ± 1.87 |
| Subtidal | 1.17 ± 0.05 | 0.37 ± 0.13 | 1694 ± 222 | 94.6 | 3471 ± 408 | 11.01 ± 1.73 |
| Reference | 1.21 ± 0.03 | 0.27 ± 0.02 | 923 | 103.4 | 2962 ± 796 | 7.84 ± 1.89 |
| **Kennedy Cove** | | | | | | |
| Intertidal | 1.22 ± 0.18 | 0.46 ± 0.25 | 1280 ± 273 | 124.9 | 2554 ± 746 | 14.86 ± 8.00 |
| Subtidal | 1.36 ± 0.17 | 0.48 ± 0.26 | 2106 ± 345 | 113.5 | 3621 ± 539 | 22.26 ± 11.35 |
| Reference | 1.40 ± 0.06 | 0.29 ± 0.07 | 1027 | 103.7 | 2764 ± 657 | 9.54 ± 3.50 |
