# Supplementary material for: Low blue carbon storage in eelgrass (Zostera marina) meadows on the Pacific Coast of Canada
Source: PLoS One. 2018 Jun 13;13(6):e0198348. doi: 10.1371/journal.pone.0198348 (PMC5999096; doi:10.1371/journal.pone.0198348)
Supplement: S5 Table — RP: Robert Point, GB: Grice Bay, KC: Kennedy Cove, IT: intertidal, ST: subtidal, NS: not sampled. (DOCX) [file pone.0198348.s006.docx]

**S5 Table. Ages of sediment in Robert Point, Grice Bay, and Kennedy Cove, in the intertidal, subtidal, and reference sites, estimated using the ^210^Pb Constant Rate of Supply (CRS) model.** RP: Robert Point, GB: Grice Bay, KC: Kennedy Cove, IT: intertidal, ST: subtidal, NS: not sampled

|  | **Robert Point**  **(years old)** | | **Grice Bay**  **(years old)** | | **Kennedy Cove**  **(years old)** | |
| --- | --- | --- | --- | --- | --- | --- |
| **Depth (cm)** | **Intertidal** | **Subtidal** | **Intertidal** | **Subtidal** | **Intertidal** | **Subtidal** |
| **1** | 3.17 | 3.24 | 2.31 | 2.82 | 3.0 | 2.35 |
| **2** | 6.5 | 6.5 | 4.8 | 5.7 | 6.4 | 4.5 |
| **3** | 9.9 | 10.2 | 7.2 | 8.6 | 10.0 | 7.2 |
| **4** | 13.2 | 13.6 | 9.5 | 11.5 | 13.8 | 10.2 |
| **5** | 16.7 | 17.4 | 12.0 | 14.4 | 17.2 | 13.1 |
| **6** | 20.1 | 21.3 | 14.4 | 17.3 | 20.6 | 16.4 |
| **7** | 23.6 | 25.0 | 16.8 | 20.2 | 23.8 | 19.2 |
| **8** | NS | NS | NS | NS | NS | NS |
| **9** | 31.6 | 33.0 | 21.8 | 26.2 | 29.9 | 26.1 |
| **11** | 40.2 | 41.8 | 27.0 | 32.9 | 38.3 | 32.7 |
| **13** | 50.1 | 52.2 | 32.4 | 40.0 | 47.4 | 40.9 |
| **15** | 62.8 | 63.7 | 38.2 | 47.1 | 59.7 | 49.2 |
| **17** | 76.4 | 75.6 | 43.6 | 54.5 | 74.4 | 57.6 |
| **19** | 93.2 | 90.8 | 49.6 | 61.9 | 93.3 | 67.0 |
| **21** | 114.5 | NS | 56.0 | 68.5 | 124.9 | NS |
| **22** | NS | NS | NS | NS | NS | NS |
| **23** | NS | NS | 62.5 | 75.8 | NS | 84.5 |
| **27** | NS | NS | 75.6 | 94.6 | NS | 113.5 |
| **31** | NS | NS | 94.3 | NS | NS | NS |
