# Supplementary material for: Low blue carbon storage in eelgrass (Zostera marina) meadows on the Pacific Coast of Canada
Source: PLoS One. 2018 Jun 13;13(6):e0198348. doi: 10.1371/journal.pone.0198348 (PMC5999096; doi:10.1371/journal.pone.0198348)
Supplement: S6 Table — SD: standard deviation, DW: dry weight. (DOCX) [file pone.0198348.s007.docx]

|  | **Nitrogen (% DW)** | | **C:N ratio** | |
| --- | --- | --- | --- | --- |
| **Zone** | **Average** | **SD** | **Average** | **SD** |
| **Robert Point** | | | | |
| Intertidal | 0.024 | 0.15 | 10.29 | 2.04 |
| Subtidal | 0.027 | 0.021 | 8.62 | 2.14 |
| **Grice Bay** | | | | |
| Intertidal | 0.042 | 0.008 | 8.30 | 1.65 |
| Subtidal | 0.043 | 0.007 | 8.27 | 0.61 |
| **Kennedy Cove** | | | | |
| Intertidal | 0.077 | 0.016 | 12.01 | 2.23 |
| Subtidal | 0.090 | 0.010 | 11.07 | 0.21 |

**S6 Table. Average nitrogen content (%N) and carbon:nitrogen ratio (C:N) of sediment in the intertidal and subtidal zones of Robert Point, Grice Bay and Kennedy Cove.** SD: standard deviation, DW: dry weight
